# Tomato root transcriptome response to a nitrogen-enriched soil patch

**DOI:** 10.1186/1471-2229-10-75

**Published:** 2010-04-27

**Authors:** Daniel R Ruzicka, Felipe H Barrios-Masias, Natasha T Hausmann, Louise E Jackson, Daniel P Schachtman

**Affiliations:** 1 Donald Danforth Plant Science Center, 975 N Warson Rd., St. Louis MO 63132 USA; 2 Department of Land, Air, and Water Resources, University of California, 1 Shields Avenue, Davis CA 95616 USA

## Abstract

**Background:**

Nitrogen (N), the primary limiting factor for plant growth and yield in agriculture, has a patchy distribution in soils due to fertilizer application or decomposing organic matter. Studies in solution culture over-simplify the complex soil environment where microbial competition and spatial and temporal heterogeneity challenge roots' ability to acquire adequate amounts of nutrients required for plant growth. In this study, various ammonium treatments (as ^15^N) were applied to a discrete volume of soil containing tomato (*Solanum lycopersicum*) roots to simulate encounters with a localized enriched patch of soil. Transcriptome analysis was used to identify genes differentially expressed in roots 53 hrs after treatment.

**Results:**

The ammonium treatments resulted in significantly higher concentrations of both ammonium and nitrate in the patch soil. The plant roots and shoots exhibited increased levels of ^15^N over time, indicating a sustained response to the enriched environment. Root transcriptome analysis identified 585 genes differentially regulated 53 hrs after the treatments. Nitrogen metabolism and cell growth genes were induced by the high ammonium (65 μg NH_4_^+^-N g^-1 ^soil), while stress response genes were repressed. The complex regulation of specific transporters following the ammonium pulse reflects a simultaneous and synergistic response to rapidly changing concentrations of both forms of inorganic N in the soil patch. Transcriptional analysis of the phosphate transporters demonstrates cross-talk between N and phosphate uptake pathways and suggests that roots increase phosphate uptake via the arbuscular mycorrhizal symbiosis in response to N.

**Conclusion:**

This work enhances our understanding of root function by providing a snapshot of the response of the tomato root transcriptome to a pulse of ammonium in a complex soil environment. This response includes an important role for the mycorrhizal symbiosis in the utilization of an N patch.

## Background

Nitrogen (N) is often a primary limiting factor for plant growth and yield in agriculture. Applications of N in conventional agriculture include fertilizer banding to the side of the plants, broadcasting on the surface of soil, and anhydrous ammonia injections. These N application methods as well as localized microbial turnover of organic N can result in spatial and temporal heterogeneity (patchiness) of soil N resulting in non-uniform availability to plant roots. Furthermore, the rapid immobilization and nitrification of N additions by soil microbes can quickly alter N availability to the root [1-3]. Roots respond to localized nutrient patches by up-regulating ion transporters and by the proliferation of new roots into the patch to capture the additional N [4-6]. Mycorrhizal fungi provide plants with an additional mechanism to explore the soil and capture nutrients from enriched regions, increasing nutrient uptake potential [7,8].

Plant roots predominantly acquire N from the rhizosphere as inorganic ammonium (NH_4_^+^) or nitrate (NO_3_^-^), and subsequently assimilate intracellular NH_4_^+ ^into amino acids [9,10]. Roots sense and respond to changes in internal and external N status, which includes the regulation of gene expression, metabolism, and further N uptake and assimilation [11,12]. High and low affinity N transport systems in roots allow plants to maximize uptake depending on soil N availability. High affinity transport systems are induced or activated under conditions where soil N availability is reduced (1 μM to 0.5 mM), while low affinity transport systems (active above ~0.5 mM N) may be constitutively expressed and transport N into the plant when soil N concentrations are high [13]. Members of the NH_4_^+ ^transporter (AMT) gene family [14] transport NH_4_^+ ^across the plasma membrane of root epidermal cells where it may be locally assimilated [15]. Intracellular NH_4_^+ ^is assimilated into glutamine and glutamate via the N-regulated glutamine synthetase (GS) and glutamate synthase (GOGAT) enzymes [16]. The NO_3_^- ^transporters (NRT) are also encoded by a large gene family [17]. The NO_3_^- ^taken up by roots can be translocated to the shoot or reduced in roots to nitrite (NO_2_^-^) and then NH_4_^+ ^via N-regulated NO_3_^- ^reductase and NO_2_^- ^reductase [13,16]. Both AMTs and NRTs exhibit complex gene regulation patterns in response to various forms and concentrations of N. These transporters are regulated by internal and external N and provide roots with a mechanism to mount a coordinated response that may serve to increase N acquisition and metabolism [11,18,19].

Recent studies have moved beyond examining expression changes of single genes or gene families to studying global changes in plant gene regulation by nutrients [19-22]. Microarray analyses of Arabidopsis and tomato roots subjected to increased NO_3_^- ^identified hundreds of differentially regulated genes whose functions included N metabolism, cell growth, and transcription [19,21]. However, most genomics studies on plant nutrient metabolism have utilized hydroponic-grown non-mycorrhizal plants, potentially limiting their translatability to our understanding of roots response in soil where nutrients are not distributed uniformly and inorganic N is being transformed. In this report we used molecular tools to study root response to the application of known concentrations of NH_4_^+ ^in a well defined region of the soil. Our aim was to characterize how roots respond to a nutrient patch in natural soils where complex ecological processes are occurring including mycorrhizal colonization and the microbial transformation of NH_4_^+ ^to NO_3_^-^. This approach is in contrast to previous studies [19-22] that have used hydroponics to study responses to NH_4_^+ ^or NO_3_^- ^singly rather than a dynamic situation which is more relevant to agriculture or natural ecosystems where NH_4_^+ ^is rapidly transformed to NO_3_^-^. We report on ^15^N uptake and translocation and the coordinated changes in gene expression patterns in mycorrhizal roots following a localized pulse of NH_4_^+ ^as NO_3_^- ^gradually became more available.

## Results

### Soil N, plant status, and plant N uptake

**Figure 1:**
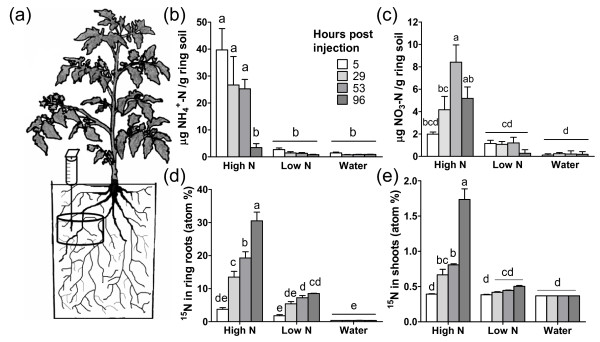
**Ammonium treatments altered soil nitrogen dynamics and plant N uptake**. (a) Diagram of pot-grown tomato plants where water, low NH_4_^+^(6.5 μg NH_4_^+^-N g^-1 ^soil), and high NH_4_^+ ^(65 μg NH_4_^+^-N g^-1 ^soil) treatments were injected into buried soil rings. (b) and (c) NH_4_^+^-N and NO_3_^-^-N concentrations per gram of patch soil in the three treatment groups at 5, 29, 53, and 96 hrs after injection. (d) and (e) atom percent ^15^N in roots and shoots 5, 29, 53, and 96 hrs after injection of the three treatment groups with ^15^N labeled NH_4_^+ ^fertilizer. Data represent the mean ± SEM of 3 biological replicates. Within each graph, means followed by different letters are significantly different from one another at P < 0.05 (two-way ANOVA with Tukey-Kramer HSD test).

Previous work showed that the field soil used for this study contained low concentrations of inorganic N, high soil N mineralization potential, moderate mycorrhizal colonization of tomato roots, and very few changes in the soil food web after nutrient addition [8]. In order to create a nutrient patch and recover roots that were directly exposed to the treatment, pots were prepared with a soil root in-growth core (ring) buried 5 cm below the soil surface and subsequently referred to as the patch (Figure 1a). A pulse of ^15^NH_4_^+ ^was injected into the soil patch to simulate the short-term effects of soil inorganic N spatial heterogeneity. The experimental design consisted of the addition of a high NH_4_^+ ^treatment (65 μg ^15^NH_4_^+^-N g^-1 ^soil) 100-fold higher than ambient NH_4_^+ ^levels, a low NH_4_^+ ^treatment (6.5 μg ^15^NH_4_^+^-N g^-1 ^soil) 10-fold higher than ambient NH_4_^+ ^levels, and a water treatment to control for any potential mobilization of nutrients that occurs when soil moisture is increased.

The NH_4_^+ ^treatments increased the soil inorganic N concentrations in the patch soil, simulating heterogeneous soil patches. In the high NH_4_^+ ^treatment group, the highest soil NH_4_^+ ^concentration (39.7 μg NH_4_^+^-N g^-1 ^dry soil) was measured at the time of the first sampling which was 5 hrs after injection. At 53 hrs after treatment it remained significantly higher than controls (25.3 μg NH_4_^+^-N g^-1 ^dry soil) (Fig 1b), and by 96 hrs, decreased to 3.5 μg NH_4_^+^-N g^-1 ^dry soil due to microbial transformations and/or plant uptake of the added NH_4_^+^. In the high NH_4_^+ ^treatment rings, soil NO_3_^- ^concentrations were above ambient levels within 29 hours, indicating nitrification of NH_4_^+ ^(Figure 1c). Patch soil NO_3_^- ^concentrations increased over the first 53 hrs after injection of the high NH_4_^+ ^treatment. In the low NH_4_^+ ^treatment, soil NH_4_^+ ^and NO_3_^- ^concentrations were similar to the water controls.

Over the course of the experiment, plant shoot growth was unaffected by treatment (P = 0.78). Percent total N (mean ± SE) in the shoots was 2.29% ± 0.08 at the time of treatment and unaffected by the N treatments (P = 0.85). Percent phosphate (P) (mean ± SE) in the shoots was 0.18% ± 0.002 and was unaffected by the N treatments. Roots were significantly colonized by arbuscular mycorrhizal fungi as measured by microscopic counting (33.5% ± 8.4, mean ± SE) and fungal transcript analysis (data not shown).

To test whether the NH_4_^+ ^treatments resulted in measurable ^15^N uptake and translocation, atom percent ^15^N was assayed in roots and leaves (Figure 1d and 1e). Within 29 hrs, patch roots and leaves from high NH_4_^+ ^plants contained increased amounts of ^15^N compared to naturally occurring ^15^N levels in the water control. The amount of ^15^N in these tissues continued to increase over time. In the patch roots from the low NH_4_^+ ^treatment, atom % ^15^N was not higher compared to the water controls until 53 hrs after injection, and there was no significant enrichment detected in leaves of the low NH_4_^+ ^treatments at any time point. There were significantly higher concentrations of ^15^N in the high NH_4_^+ ^treatment roots and leaves compared to the low NH_4_^+ ^treatment samples at multiple time points, further confirming a physiological difference between these treatments.

### Microarray analysis of patch root transcription

**Figure 2:**
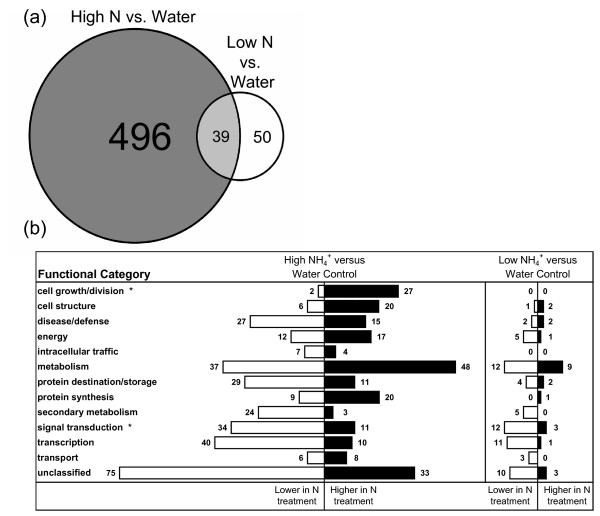
**Classification of nitrogen treatment-regulated genes into defined functional categories**. (a) Venn diagram displaying the number of genes identified in either or both the high NH_4_^+ ^vs. water and low NH_4_^+ ^vs. water comparisons. (b) Affymetrix probeset sequences were matched to publicly available Genbank accession identifications and categorized according to tomato or Arabidopsis orthologue gene annotations. Differential expression was analyzed at 53h post treatment. Black bars represent the number of genes more highly expressed in the NH_4_^+ ^treatment compared to the water treatment, and white bars represent the number more highly expressed in the water treatment compared to the NH_4_^+ ^treatment. Asterisks indicate a significant difference in the proportion of genes in those functional categories between the high NH_4_^+ ^vs. water and low NH_4_^+ ^vs. water comparisons.

Affymetrix Tomato GeneChips were used to analyze the root transcriptome at 53 hrs post-treatment; when ^15^N enrichment levels were detected in roots from both high and low NH_4_^+ ^treatment groups. Array analysis detected expression of 5822 of the 9524 transcripts contained on the tomato genechip. Statistical analyses identified 585 genes that were significantly altered in expression among the three treatment groups (Additional file 1). The high NH_4_^+ ^treatment resulted in a much larger transcriptome response than the low NH_4_^+ ^treatment, with 535 genes differentially expressed between the high NH_4_^+ ^treatment and water control, compared to 89 genes with different expression levels between low NH_4_^+ ^treatment and water control (Figure 2a). While there were many differences between the regulated genes under high or low NH_4_^+ ^vs. water control treatments, 39 genes were identified as differentially regulated in both comparison groups, and all 39 were similarly regulated by the high and low NH_4_^+ ^treatments compared to water (Figure 2a and Additional file 2).

We annotated and categorized nearly 80% of the 585 differentially regulated genes into putative functional classes. The NH_4_^+ ^treatments resulted in the differential regulation of genes in a wide range of functional categories (Figure 2b). In every functional category, the high NH_4_^+ ^vs. water comparison contained a significantly higher number of genes compared to the low NH_4_^+ ^vs. water comparison, further indicating that the high NH_4_^+ ^treatment resulted in a larger scale transcriptional response. Fisher's exact test was used to determine whether the gene lists from the [high NH_4_^+ ^vs. water] or [low NH_4_^+ ^vs. water] comparisons were enriched for different functional categories compared to one another, indicating a unique type of response to the two NH_4_^+ ^treatments. Significantly more cell growth and division genes were identified in the high NH_4_^+ ^vs. water comparison (29 out of 535, 5.4%) than the low NH_4_^+ ^vs. water comparison (0 out of 89, 0%) (P = 0.0245). Conversely, significantly more signal transduction genes were identified in the low NH_4_^+ ^vs. water comparison (15 out of 89, 16.9%) than the high NH_4_^+ ^vs. water comparison (45 out of 535, 8.4%) (P = 0.0187).

A significant proportion (39 out of 89, 43.8%) of the genes in the low NH_4_^+ ^vs. water comparison were similarly regulated in the high NH_4_^+ ^vs. water comparison (Additional file 2), and included N assimilation and metabolism genes such as glutamine synthetase and tryptophan synthase. Eight of the remaining 50 (16%) genes uniquely identified only in the low NH_4_^+ ^vs. water comparison function in sugar metabolism including glucosyl hydrolase, trehalose 6-phosphate synthase, low affinity sucrose transporter *SUT4*, isocitrate lyase, and glucose 6-phosphate translocator.

### Identification of genes regulated among the N treatments

Multiple genes in the N transporters, N metabolism, and amino acid metabolism subcategories were highly responsive to the NH_4_^+ ^treatments. The high NH_4_^+ ^treatment resulted in the increased expression level of N transporter, assimilation, and metabolism genes in roots including NH_4_^+ ^transporter *AMT2*, NO_3_^- ^transporter *NRT2.1*, nitrite reductase *Nii2*, glutamine synthetases *GS *(chloroplastic) and *GTS1 *(cytosolic), and NADH-dependant glutamate synthase *GLT1 *(GOGAT) (Table 1). Additional genes including peptide transporter 1 (*LeOPT1*), NH_4_^+ ^transporter 1 (*AMT1*), and nitrilase (*NIT4*) exhibited lower expression levels in the high NH_4_^+ ^treatment roots compared to water control samples. The nitrate transporters *NRT2.3 *and *NRT3.2 *were found on the array but were not differentially regulated among the treatment groups. Multiple amino acid metabolism genes were regulated by the N treatments including higher expression levels of two aspartate aminotransferases, an alanine aminotransferase, and a tryptophan synthase (Table 1). Lower expression levels of chorismate synthase 2 and alpha-aminoadipic semialdehyde synthase were detected (Table 1). A tomato MADS-Box transcription factor similar to the Arabidopsis N-starvation response transcription factor ANR1 was expressed 3.26-fold higher in the water control treatment compared to high NH_4_^+ ^samples (Table 1).

**Table 1:** Differentially regulated nitrogen assimilation and metabolism genes.

Probe Set ID	Putative Annotation	Fold Change (High N vs. water)	P-value	Fold Change (Low N vs. water)	P-value
Les.224.1.S1_at	glutamine synthetase	**16.69**	0.002	**6.31**	0.024

Les.2360.1.S1_at	nitrite reductase	**4.25**	0.044	1.39	0.364

Les.3640.1.S1_at	ammonium transporter	**4.07**	0.045	1.24	0.638

Les.2884.1.S1_at	glutamine synthetase	**3.23**	0.044	1.39	0.365

Les.28.2.S1_a_at	nitrate transporter 2.1	**2.61**	0.082	1.42	0.402

Les.987.1.A1_at	aspartate aminotransferase	**2.29**	0.050	-1.02	0.943

Les.987.3.S1_at	aspartate aminotransferase	**2.18**	0.044	-1.34	0.216

Les.5163.1.S1_at	dicarboxylate transport	**1.85**	0.075	1.99	0.105

Les.899.1.S1_at	NADH-dependent glutamate synthase	**1.76**	0.064	-1.05	0.834

Les.3626.1.S1_at	alanine aminotransferase	**1.71**	0.092	1.36	0.246

Les.2756.1.A1_at	tryptophan synthase-related	**1.53**	0.095	**2.01**	0.078

Les.231.1.S1_at	O-acetyl(thiol)serine lyase	1.32	0.136	**1.63**	0.088

Les.3660.1.S1_at	chorismate synthase 2	**-1.73**	0.061	-1.36	0.180

Les.299.1.S1_at	peptide transporter 1	**-1.75**	0.093	-1.37	0.254

Les.797.1.S1_at	ammonium tranporter 1	**-1.80**	0.065	-1.09	0.700

Les.3289.1.S1_at	g-aminobutyrate transaminase subunit precursor	**-1.84**	0.053	-1.76	0.105

Les.7.1.S1_at	homogentisate 1,2-dioxygenase HGO	**-1.98**	0.092	-1.54	0.214

Les.1493.1.S1_at	nitrilase	**-2.02**	0.098	-1.65	0.192

LesAffx.3336.1.S1_at	cystathionine beta-synthase domain protein	-2.15	0.108	**-3.60**	0.078

Les.3071.1.S1_at	alpha-aminoadipic semialdehyde synthase	**-2.27**	0.067	-2.35	0.105

Les.5024.1.S1_at	ANR1-like MADS-box transcription factor	**-3.26**	0.067	-2.28	0.156

Probe Set ID; Affymetrix identifier for each microarray probeset. Putative Annotation; functional annotation based on tomato protein function or function of Arabidopsis orthologues identified with BLAST searches. Fold Change; linear fold changes (bold values significant at False Discovery Rate (FDR) adjusted P-value < 0.10). High N = added 65 μg ^15^NH_4_-N per gram soil, low N = added 6.5 μg ^15^NH_4_-N per gram soil. Probe Set IDs Les.987.1.A1_at and Les.987.3.S1_at represent the same genes.

**Figure 3:**
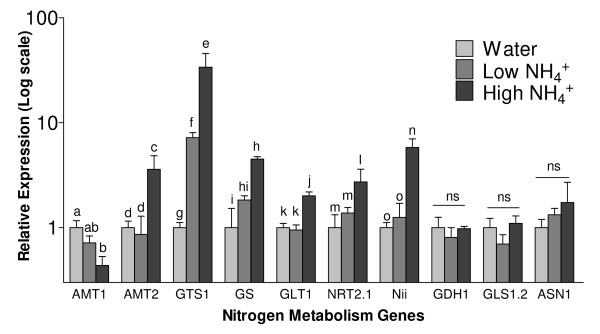
**qRT-PCR analysis of key nitrogen metabolism genes**. Expression levels of NH_4_^+ ^transporters AMT1 and AMT2, glutamine synthetases GTS1 and GS, NO_3_^- ^transporter NRT2.1, nitrite reductase Nii, NADH-dependant glutamate synthase GLT1 (GOGAT), glutamate dehydrogenase GDH1, ferrodoxin-dependant glutamate synthase GLS1.2 (GOGAT), and asparagine synthetase ASN1 in patch roots harvested 53 hrs after high (65 μg NH_4_^+^-N g^-1 ^soil), low (6.5 μg NH_4_^+^-N g^-1 ^soil), or water control treatments. Relative quantity was calculated using the ΔΔCT method with actin (LeACT) as the reference control, and the water control group normalized to 1. For a given gene, means followed by different letters are significantly different from one another at P < 0.05 (one way ANOVA).

We used qRT-PCR to quantify the expression of key N metabolism genes to confirm the accuracy of the array results. The qRT-PCR results largely agreed with both the direction and magnitude of expression levels across the three treatments, including three genes that were not significantly different by array or qRT-PCR (glutamate dehydrogenase *GDH1*, ferrodoxin-dependant glutamate synthase *GLS1.2 *(GOGAT), and asparagine synthetase *ASN1*) (Figure 3).

Microarrays identified genes that were differentially regulated among the NH_4_^+ ^treatments from other functional categories including cell growth/division, cell wall biosynthesis, and stress/defense response, and sulfur metabolism (Figure 2b, Tables 2, 3, and 4, and Additional file 1). In the cell growth and division category, there were significantly more genes induced by the high NH_4_^+ ^treatment vs. water control than repressed by the high NH_4_^+ ^treatment (27 out of 29 cell growth and division genes induced by high NH_4_^+^, P < 0.001) (Table 2). This category of genes included multiple cyclins, histones, and other growth factors. A similar pattern was observed for cell wall biosynthesis genes encoding multiple pectinesterases, expansins, endo-xyloglucan transferases, and cellulose synthase (18 out of 20 induced by high NH_4_^+^, P < 0.001) (Table 3).

**Table 2:** Differentially regulated cell growth and division genes.

Probe Set ID	Putative Annotation	Fold Change (High N vs. water)	P-value
Les.5082.1.S1_at	cdc20 cell cycle regulator	**3.46**	**0.044**

Les.2170.1.A1_at	cyclin	**2.89**	**0.062**

Les.5789.1.S1_at	histone 3	**2.74**	**0.045**

Les.103.1.S1_at	cyclin A1	**2.65**	**0.044**

Les.3009.3.A1_at	histone HTA12	**2.52**	**0.053**

Les.3713.1.S1_at	B2-type cyclin dependent kinase	**2.48**	**0.044**

Les.677.1.S1_at	histone H2AX	**2.33**	**0.053**

Les.677.2.A1_at	histone H2AX	**2.31**	**0.061**

Les.5740.1.S1_at	replicon protein A	**2.28**	**0.044**

LesAffx.66157.1.S1_at	mitotic arrest deficient-like	**2.21**	**0.068**

LesAffx.19390.1.S1_at	cyclin	**2.16**	**0.044**

Les.3209.1.S1_at	histone H4 replacement isoform	**2.11**	**0.044**

Les.3090.1.S1_at	histone H3	**2.08**	**0.044**

Les.3555.1.S1_at	histone H2B-2	**2.00**	**0.044**

Les.4603.1.S1_at	histone H3	**1.96**	**0.044**

Les.5283.1.S1_at	minichromosome maintenance protein	**1.83**	**0.084**

Les.4439.1.S1_at	histone H3	**1.80**	**0.053**

Les.4978.1.S1_at	DNA-dependant ATPase	**1.77**	**0.091**

Les.4442.1.S1_s_at	histone H2B-1	**1.73**	**0.044**

LesAffx.57438.1.S1_at	nucleosome chromatin assembly factor	**1.72**	**0.081**

Les.3209.2.A1_at	histone H4 replacement isoform	**1.72**	**0.062**

Les.4831.1.S1_at	nucleosome chromatin assembly factor	**1.64**	**0.053**

Les.4539.1.S1_a_at	histone H4	**1.57**	**0.091**

LesAffx.2226.2.A1_at	ribonucleotide reductase-like	**1.57**	**0.091**

Les.4564.1.S1_at	microtubule associated protein	**1.54**	**0.088**

Les.2989.1.S1_at	histone HTA7	**1.51**	**0.044**

Les.4539.2.S1_at	histone H4	**1.50**	**0.091**

Les.3009.2.S1_at	histone HTA12	**1.48**	**0.066**

Les.4940.1.S1_at	cyclin	**-1.56**	**0.088**

Les.3563.1.S1_at	ER auxin binding protein 1	**-1.74**	**0.082**

Probe Set ID; Affymetrix identifier for each microarray probeset. Putative Annotation; functional annotation based on tomato protein function or function of Arabidopsis orthologues identified with BLAST searches. Fold Change; linear fold changes (bold values significant at False Discovery Rate (FDR) adjusted P-value < 0.10). Probe Set IDs representing the same genes include (Les.3009.3.A1_at and Les.3009.2.S1_at), (Les.677.1.S1_at and Les.677.2.A1_at), (Les.3209.1.S1_at and Les.3209.2.A1_at) and (Les.4539.1.S1_a_at and Les.4539.2.S1_at).

**Table 3:** Differentially regulated cell wall metabolism genes.

Probe Set ID	Putative Annotation	Fold Change (High N vs. water)	P-value	Fold Change (Low N vs. water)	P-value
Les.3273.1.S1_at	cell wall-plasma membrane linker	**5.98**	**0.053**	1.63	0.424

LesAffx.846.2.S1_at	pectinacetylesterase	**3.22**	**0.080**	-1.44	0.458

Les.3733.1.S1_at	expansin	**3.20**	**0.067**	1.45	0.414

Les.3590.1.S1_at	endo-xyloglucan transferase	**3.12**	**0.081**	-1.33	0.565

LesAffx.4617.1.A1_at	pectinesterase	**3.10**	**0.063**	-1.41	0.417

Les.2316.1.S1_at	cellulose synthase isomer	**3.01**	**0.067**	1.88	0.193

Les.2189.1.S1_at	pectinesterase	**2.51**	**0.070**	-1.07	0.868

Les.218.3.S1_at	pectinesterase	**2.07**	**0.094**	-1.14	0.710

Les.1604.1.A1_at	cobra-like4 phytocheletin synthase	**1.91**	**0.096**	1.51	0.219

Les.369.1.S1_at	expansin	**1.87**	**0.074**	1.48	0.186

LesAffx.69659.1.S1_at	chitinase class IV	**1.82**	**0.063**	1.65	0.127

Les.5233.1.S1_at	pectinesterase	**1.76**	**0.089**	1.06	0.829

Les.218.1.S1_at	pectinesterase	**1.73**	**0.096**	1.27	0.362

Les.3523.1.S1_at	polygalacturonase	**1.72**	**0.096**	1.17	0.551

Les.4739.1.S1_at	UDP-glucose:protein transglucosylase	**1.70**	**0.048**	1.28	0.196

Les.109.1.S1_at	beta-galactosidase	**1.61**	**0.065**	-1.02	0.944

Les.4707.1.S1_at	pectate lyase	**1.61**	**0.061**	**1.67**	**0.093**

Les.2590.2.A1_at	endo-xyloglucan transferase A2-like	**1.40**	**0.085**	-1.05	0.743

Les.4523.1.S1_at	xyloglucan endotransglucosylase-hydrolase	**-2.53**	**0.079**	-1.58	0.265

Les.4652.1.S1_at	esterase/lipase/thioesterase	**-3.76**	**0.063**	-1.78	0.271

Probe Set ID; Affymetrix identifier for each microarray probeset. Putative Annotation; functional annotation based on tomato protein function or function of Arabidopsis orthologues identified with BLAST searches. Fold Change; linear fold changes (bold values significant at False Discovery Rate (FDR) adjusted P-value < 0.10). Probe Set IDs Les.218.1.S1_at and Les.218.3.S1_at represent the same genes.

**Table 4:** Differentially regulated stress and defense response genes.

Probe Set ID	Putative Annotation	Fold Change (High N vs. water)	P-value	Fold Change (Low N vs. water)	P-value
Les.2287.3.A1_at	TAS14 peptide dehydrin	**-24.08**	**0.053**	**-40.75**	**0.078**

Les.5957.1.S1_at	lactoylglutathione lyase	-6.87	0.111	**-17.15**	**0.078**

Les.293.1.S1_at	hydroxyacylglutathione hydrolase	-4.66	0.513	**-4.44**	**0.078**

Les.23.1.S1_at	glutathione S-transferase	-4.54	0.569	**-3.07**	**0.078**

Les.124.1.S1_at	glutathione transferase	**-4.38**	**0.044**	-2.54	0.107

Les.5100.1.S1_at	type I small heat shock protein	**-3.85**	**0.044**	-2.20	0.107

Les.4789.1.S1_at	pathogenesis-related protein	**-3.47**	**0.062**	-2.12	0.115

Les.5341.1.S1_at	pathogen responsive alpha-dioxygenase 2	**-2.76**	**0.098**	-1.85	0.131

Les.1645.1.A1_at	pathogenesis-related chitin-binding protein	**-2.44**	**0.065**	-1.74	0.133

Les.4004.1.S1_a_at	pathogenesis related PR5-like protein	**-2.26**	**0.070**	-1.59	0.143

Les.5098.1.S1_at	early responsive to dehydration 7-like	**-2.20**	**0.064**	-1.42	0.152

LesAffx.47187.1.S1_at	responsive to dehydration 22-like	**-2.12**	**0.058**	-1.34	0.165

Les.5103.1.S1_at	pathogenesis-related protein 1 like	**-2.04**	**0.062**	-1.33	0.168

Les.3151.1.S1_at	universal stress protein	**-1.99**	**0.084**	-1.33	0.168

Les.253.1.S1_at	pathogenesis related protein 1-like	**-1.95**	**0.044**	-1.29	0.229

Les.4910.1.S1_at	stress enhanced protein 2-like	**-1.93**	**0.085**	-1.26	0.232

Les.1498.1.S1_at	dehydration responsive	**-1.87**	**0.096**	-1.25	0.289

Les.208.1.S1_at	glutathione S-transferase	**-1.72**	**0.062**	-1.22	0.340

Les.2657.1.S1_at	rare cold inducible protein-like	**-1.70**	**0.095**	-1.20	0.377

Les.3194.1.S1_at	universal stress protein	**-1.62**	**0.088**	-1.16	0.386

Les.5128.1.S1_at	responsive to dehydration 22-like	**-1.61**	**0.044**	-1.14	0.454

Les.3276.3.S1_at	monocysteinic thioredoxin	**-1.56**	**0.093**	-1.11	0.509

Les.252.1.S1_at	wound-responsive protein-related	**-1.50**	**0.077**	-1.11	0.524

Les.54.1.S1_at	sulfiredoxin	**1.10**	**0.044**	-1.10	0.537

Les.248.2.A1_at	glutathione S-transferase	**1.14**	**0.095**	-1.08	0.796

Les.384.1.A1_at	thaumatin-like pathogenesis-related PR-5 like protein	**1.42**	**0.070**	-1.07	0.850

Les.4307.1.S1_at	early responsive to dehydration 3-like	**1.47**	**0.053**	-1.05	0.870

Les.1394.1.A1_at	heat shock factor binding protein 1	**1.82**	**0.063**	1.22	0.185

Les.3593.1.S1_at	heat shock protein	**2.13**	**0.044**	1.45	0.204

Les.2409.1.S1_at	dnaJ related molecular chaperone	**2.30**	**0.050**	1.57	0.537

LesAffx.43379.1.S1_at	dnaJ homologue 3	**2.71**	**0.052**	1.64	0.623

LesAffx.71535.1.S1_at	heat shock protein	**2.81**	**0.077**	1.72	0.639

Les.641.1.S1_at	dnaJ heat shock protein	**3.01**	**0.067**	1.95	0.746

LesAffx.66226.2.S1_at	cold-regulated plasma membrane 1 protein	**3.28**	**0.044**	1.97	0.180

Les.5158.1.S1_at	dehydration response element B1A	**3.36**	**0.092**	1.99	0.218

LesAffx.59336.1.S1_at	response to desiccation 26-like transcription factor	**3.59**	**0.082**	2.25	0.231

Probe Set ID; Affymetrix identifier for each microarray probeset. Putative Annotation; functional annotation based on tomato protein function or function of Arabidopsis orthologues identified with BLAST searches. Fold Change; linear fold changes (bold values significant at False Discovery Rate (FDR) adjusted P-value < 0.10).

The NH_4_^+ ^treatments caused various transcriptional responses in multiple stress and defense-response genes, and genes in this functional category were some of the most differentially regulated genes of the experiment (Table 4). Genes in certain stress and defense response subcategories exhibited similar transcriptional responses including glutathione metabolism genes (6 out of 7 higher expression in water samples), pathogen response factors (5 out of 6 higher expression in water samples), and heat shock proteins (6 out of 7 higher expression in high NH_4_^+ ^samples).

### Phosphate transporters

**Figure 4:**
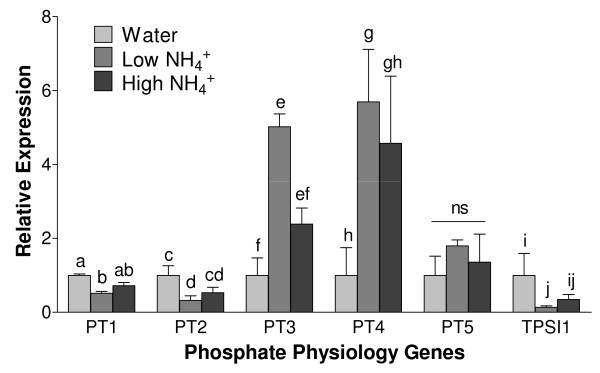
**qRT-PCR analysis of phosphate physiology genes**. Expression levels of the tomato phosphate transporters and phosphate starvation response gene TPSII in patch roots harvested 53 hrs after high (65 μg NH_4_^+^-N g^-1 ^soil), low (6.5 μg NH_4_^+^-N g^-1 ^soil), or water control treatments. Relative quantity was calculated using the ΔΔCT method with actin (LeACT) as the reference control, and the water control group normalized to 1. For a given gene, means followed by different letters are significantly different from one another at P < 0.05 (one way ANOVA).

The patch roots in this study were colonized by arbuscular mycorrhizal (AM) fungi therefore we examined the transcriptome data to determine whether the NH_4_^+ ^treatments might affect known symbiosis processes including the transcriptional regulation of the phosphate transporters (PTs). The tomato PTs group into mycorrhiza-specific, mycorrhiza-induced, and nonspecific root expression patterns, and are indicators of Pi status and the mycorrhizal symbiosis [23-26]. The Affymetrix tomato genechip only includes nonspecific phosphate transporter 1 (*PT1*), and it was 1.6-fold higher in the water samples compared to the low N treatment (P = 0.004). To test whether the NH_4_^+ ^treatments resulted in the differential regulation of the other PT family genes, we assayed the expression of the tomato phosphate transporters *PT1*, *2*, *3*, *4*, and *5 *with qRT-PCR (Figure 4). Expression of nonspecific *PT1 *and *PT2 *were 1.9- and 3.1- fold higher in the water samples compared to the low N treatment samples (P = 0.003 and 0.046, respectively). Mycorrhiza-induced *PT3 *and mycorrhiza-specific *PT4 *expression levels were 5.0- and 5.7- fold higher, respectively, in the low NH_4_^+ ^treatment group compared to the water control samples (P = 0.014 and 0.045, respectively). Mycorrhiza-induced *PT5 *expression was not different among the treatments. The expression level of the phosphate starvation-induced tomato gene *TPSI1 *was 7.5-fold higher in the water samples compared to the low NH_4_^+ ^treatment (P = 0.019). Similar trends were found for the high NH_4_^+ ^treatments compared to water controls. We analyzed shoot Pi levels to test whether the alterations in *PT *gene expression correlated with or resulted in changes in shoot total Pi levels, but they not significantly different across the treatments.

## Discussion

Previous studies have reported the transcriptional regulation of genes in diverse functional groups including metabolism, energy, cell growth, and transcription/translation in response to N nutrition as NO_3_^- ^or NH_4_^+ ^[19-22]. However, these have utilized hydroponics systems that do not necessarily reflect the rhizosphere environment encountered by plant roots in agricultural and natural ecosystems. Roots grown in solution culture systems do not compete with soil microbes for nutrients, and the concentrations of nutrients in solution are more uniform both spatially and temporally. Fertilizer application and soil processes in conventional and organic farming result in the formation of heterogeneous soil nutrient patches [4], and plant utilization of N patches depends on roots rapidly sensing and response to the local enrichment of nutrients where they are in competition with soil microbial N assimilation and nitrification, leaching, and denitrification [2,3,27,28].

To better understand plant root response to a localized and dynamic inorganic N soil patch, we utilized an experimental design that simulated a more realistic patch environment. The buried ring created the spatial attributes of an N patch by ensuring that harvested roots were localized to the NH_4_^+ ^treatment patch. Measurements of soil NH_4_^+ ^and NO_3_^- ^levels confirmed dynamic soil transformations of available N by 53 hrs when we sampled the roots for microarray analysis. After 96 hrs, we observed a trend of decreasing NH_4_^+ ^and NO_3_^- ^soil levels, indicating plant and microbial uptake of both NH_4_^+ ^and NO_3_^- ^[1,9]. Furthermore, estimates of the % recovery of applied ^15^N in shoots from high and low NH_4_^+ ^treatment groups after 96 hrs (22% and 21%, respectively) support the assertion that roots faced significant competition for N in the soil environment.

The rapid N uptake observed in this study demonstrates the ability of tomato roots to quickly capture fertilizer in soil patches [29,30]. The roots that encountered the high NH_4_^+ ^treatment took up and translocated significantly more ^15^N from the patch than the low NH_4_^+ ^treatment roots. This observation is in agreement with the larger transcriptional response to the high NH_4_^+ ^treatment including multiple nitrogen transport, assimilation, and metabolism genes. However, the relatively low % recovery suggests that actual uptake and assimilation are only a small fraction of what was initially available despite the rapid root responses.

The genes coordinately regulated in both high and low treatments may represent a conserved physiological response to different ranges of N patch conditions. The co-regulated list of genes did not include the NH_4_^+ ^or NO_3_^- ^transporters *AMT1*, *AMT2*, or *NRT2.1 *identified in the high NH_4_^+ ^vs. water control comparison. We speculate that roots in the high NH_4_^+ ^patch needed additional transporters to effectively capture the higher soil N levels, while constitutively expressed transporters were sufficient in the low NH_4_^+ ^patch. Of equal interest, however, is the set of 50 genes regulated by the low NH_4_^+ ^treatment that were not similarly regulated by the high NH_4_^+ ^treatment. This group of genes included a significant number of sugar metabolism genes for which we have no clear explanation as to the functional significance for the change in regulation. One possibility is that sugar metabolism may have been required for the production of root exudates, which can increase plant growth promoting rhizobacteria, or stimulate N cycling by microbial populations in the rhizosphere [31]. While the total number of genes regulated by the two treatments suggests that the high NH_4_^+ ^treatment caused a larger response, the responses were in part unique, and may reflect different strategies to effectively utilize the N patch. Increased N availability stimulates highly regulated root development and growth in order to efficiently scavenge and assimilate the additional soil N [32,33]. The induction of multiple histone gene family members such as histones H4, H3 and H2AX in the high NH_4_^+ ^treatment suggests an increase in DNA replication [34,35], while the increased expression of a mitotic spindle checkpoint gene, replicon protein A, multiple cyclin genes, and a putative cdc20 suggest an increase in cell division processes [36,37]. Furthermore, the induction of cell wall genes including expansins, pectinesterases, and cellulose synthase suggests an increase in cell wall biosynthesis that would be required during root growth [38]. In Arabidopsis, multiple expansins and other cell wall modification enzymes were up-regulated by NO_3_^- ^3 hrs post-treatment [20], and gene expression profiling of maize roots in early response to a NO_3_^- ^treatment identified multiple genes involved in cell growth and lateral root elongation including alpha-expansin, kinesin, and cellulose synthase [39]. These experiments imply that similar N developmental response mechanisms are conserved across maize, Arabidopsis, and tomato roots, and that the root response to an N nutrient patch includes coordinated root growth.

In this study, diverse stress response genes encoding heat-shock proteins, glutathione transferases, thioredoxin, pathogenesis-related proteins, and dehydration/desiccation responsive proteins were found to be differentially expressed among the NH_4_^+ ^treatment groups. Limiting nutrient conditions cause various stress-related responses including the up-regulation of reactive oxygen species metabolism [40]. Chronic N stress induces a range of plant stress responses which include the transcriptional regulation of numerous stress responsive genes. In Arabidopsis ~35% of the genes upregulated by a severe chronic N limitation stress were classified as response to abiotic stimulus, general stress, or oxidative stress [41]. Studies of N effects on the expression of stress response genes in rice also indicate that N limiting conditions cause the differential regulation of biotic and abiotic stress genes [42]. From their studies, Lian *et al. *postulate that this could be due to the perception of N limitation as a biotic or abiotic stress that requires a conserved set of regulated genes that play protective roles [42]. Our results suggest that the conditions in the water control samples may have initiated a stress response in roots due to an N limitation, and that the high and low NH_4_^+ ^treatments alleviated this coordinated stress response.

The complex regulation of specific NH_4_^+ ^and NO_3_^- ^transporters following the NH_4_^+ ^pulse may reflect a simultaneous and synergistic response to both NH_4_^+ ^and NO_3_^- ^in the soil patch. Both Arabidopsis *AtAMT1.1 *and tomato *LeAMT1 *exhibit increased transcript levels during N deprivation and are repressed by NO_3_^- ^and NH_4_^+ ^[43-45]. The higher expression of *LeAMT1 *under control conditions and its repression by high NH_4_^+ ^in the present study further support the idea that *LeAMT1 *is a high affinity ammonium transporter whose expression is regulated by N-limiting conditions to increase NH_4_^+ ^uptake. In two hydroponics studies, *LeAMT2 *was induced by increased concentrations of NH_4_^+ ^over the course of 24 hrs but repressed by increased concentrations of NO_3_^- ^after 24 hrs [19,44]. The higher expression of *LeAMT2 *in response to the soil N patch 53 hrs after treatment in this current study suggests that the positive effects of NH_4_^+ ^may be stronger than the long-term repressive effects of NO_3_^- ^exposure. Arabidopsis, barley and tomato NO_3_^- ^transporters *AtNRT2.1*, *HvNRT2*, and *LeNRT2.1 *were induced by NO_3_^- ^in hydroponic culture, and NH_4_^+ ^repressed *HvNRT2 *expression [13,19]. We report that *LeNRT2.1 *was induced in the high NH_4_^+ ^treatment where we measured increased NO_3_^- ^concentrations. The increased NO_3_^- ^in the patch may have induced *LeNRT2.1*, although the effects of NH_4_^+ ^alone in the absence of NO_3_^- ^on *LeNRT2.1 *remain to be tested. The complex regulation of the NH_4_^+ ^and NO_3_^- ^transporters in this study indicate that tomato roots are able to quickly sense and respond to changing concentrations of NH_4_^+ ^and NO_3_^- ^simultaneously in a localized N patch, enhancing N uptake and utilization. Moreover, growth is known to increase with co-provision of NH_4_^+ ^and NO_3_^- ^[22]. Recent studies have also reported root responses to soil glutamate that may have been available to patch roots [46]. This study highlights the ability of plant roots to simultaneously regulate multiple transporters for uptake of both forms of inorganic N as part of a plastic response strategy to quickly exploit the N pulse.

Numerous transcription factors were identified in the microarray study that may function as key regulators of a secondary response to the N enrichment. The tomato MADS-box transcription factor BT013126 shares 67% amino acid sequence similarity with Arabidopsis ANR1 and is expressed 3.26-fold higher in the water control compared to the high NH_4_^+ ^treatment (Table 1). Arabidopsis ANR1 is a key regulator of the developmental response to N in roots and is induced by N starvation and repressed by NO_3_^- ^re-supply [47,48]. Prior to the N additions, the plants were most likely N-limited as the shoot N concentration was below sufficiency levels (mean = 1.94% for water control) [49]. The expression pattern of this tomato *ANR1-like *gene in N patch roots corresponds to what was found in Arabidopsis, suggesting that its functional role to regulate root development in response to N is conserved across species and in diverse root environments.

Root responses to macronutrients N, P, potassium (K), and sulfur (S) are interconnected and may be due to the increased availability of one causing an imbalance in another. Previous studies have shown N addition to increase the expression level of S metabolism genes [21,50], which could account for the changes in S metabolism genes reported here (Additional file 1). Alternatively, these genes may have been affected by sulfate in the NH_4_^+ ^treatment, although soil S concentrations were likely sufficient for the plant. Cross talk between K and N has also been shown where K deficiency alters the transcriptional and post-transcriptional activity of various N uptake, assimilation, and metabolism genes including three nitrate transporters [51,52]. Nitrogen and phosphate metabolism have been shown to be closely linked where N uptake results in coordinated P uptake [53,54]. However, in response to a 3 hr nitrate pulse, phosphate transporter expression levels in hydroponics-grown Arabidopsis did not change [20]. In soils, mineral availability and acquisition is additionally affected by the mycorrhizal symbiosis, and previous work has linked the up-regulation of the fungal phosphate transporter *GiPT *to the presence of N [55]. In this current study, multiple phosphate transporters were regulated by the NH_4_^+ ^treatments, in contrast to the Arabidopsis findings [20]. We observed that mycorrhizal-induced PT3 and mycorrhizal-specific *PT4 *were more highly expressed when more NH_4_^+ ^was present in the soil. The *PT3 *and *PT4 *expression patterns suggest that arbuscular mycorrhizal Pi uptake may be enhanced by NH_4_^+ ^soil enrichment. Phosphate transporters *PT1 *and *PT2 *are found in both mycorrhizal and nonmycorrhizal root tissues, but are repressed in mycorrhizal roots [56-58]. The repression of *PT1 *and *PT2 *in the NH_4_^+ ^treatments in the present study further supports the conclusion that the NH_4_^+ ^treatments promoted the symbiotic Pi uptake pathway. In fact, Wang *et al*. reported that tomato *PT2 *was induced by NO_3_^- ^in hydroponic-grown non-mycorrhizal roots [19], and thus it appears that *PT2 *regulation in the current study was in response to up-regulation of the mycorrhizal Pi uptake pathway rather than soil NO_3_^- ^directly. We can speculate that the NH_4_^+ ^soil enrichment induced root growth in the nutrient patch, resulting in a localized P deficiency that promoted the mycorrhizal Pi uptake pathway. Importantly, the lower expression level of phosphate-starvation induced *TPSI1 *in the low and high NH_4_^+ ^treatment plants suggests that these roots were receiving more Pi than the water control samples [59], although this was not measured directly. This shift towards the mycorrhizal Pi uptake pathway may have resulted in increased Pi uptake, possibly as a mechanism to support N-induced growth. Our results detail a novel and complex interaction between inorganic N, the arbuscular mycorrhizal symbiosis, and the tomato phosphate transporter gene family, and suggest an important role for the symbiosis in the utilization of an N patch to increase P uptake and maintain N-induced growth.

## Conclusions

Spatially discrete NH_4_^+ ^is quickly transformed in the soil and taken up by plants, and the tomato root transcriptome reflects levels of N availability and transformations of N that occur in the soil. The dynamic regulation of both NH_4_^+ ^and NO_3_^- ^transporters in N-patch roots demonstrates that roots are able to simultaneously sense and respond to both forms of inorganic N, in ways that are likely to increase root competition with microbial immobilization, nitrification, and denitrification, and conserve N within cropping systems. The arbuscular mycorrhizal symbiosis may further increase the effective recovery of other nutrients such as P in an N patch. The strong and diverse transcriptional response to the soil N patch illustrates the utility of applying transcriptomic studies to plants growing in realistic soil environments and the key genes co-regulated under high and low N conditions in this study may serve as molecular tools for monitoring plant N status in agricultural sites for finer tuning of fertilizer application, soil microbial N processes, and ultimately, to develop more efficient agriculture methods.

## Methods

### Soil and plant material

Seeds of *Solanum lycopersicum *L. Cv. 76R [60] were surface sterilized, germinated with mist irrigation and then watered with one-tenth strength Long Ashton's solution containing N as (NH_4_)_2_SO_4 _(4 mM) and NaNO_3 _(8 mM). Plants were maintained under day/night length of 16/8 hr in UC Davis glasshouses. Seven week old seedlings were transplanted into 12-L pots containing buried rings with field-collected soil (Zamora loam, a fine silty, mixed thermic, Mollic Haploxeralfs) collected on an organically managed farm (Jim and Deborah Durst Farming in Esparto, Yolo County, California) [8]. The buried soil root in-growth cores (rings) were 7.3 cm in diameter and 4.2 cm tall (total volume 176 cm^3^) and were filled with 210 g of field soil to a final bulk density equal to that of the surrounding soil (1.2 g cm^-3^). The broad ends of the ring were covered with 1 mm plastic mesh to easily allow roots to grow up and down into the ring. The soil was passed through a 1 cm sieve before packing into pots at a bulk density of 1.2 g cm^-3^. Extractable inorganic N (mean ± standard error) was 0.20 ± 0.02 μg NH_4_^+^-N g^-1 ^dry soil and 9.7 ± 1.09 μg NO_3_-N g^-1 ^dry soil at the time the rings were prepared. An application of one tenth strength Long Ashton's solution was applied two weeks after transplanting. Soil moisture was maintained gravimetrically at 19% before and after treatment injection by weighing the pots daily, and watering to compensate for evapotranspiration water loss.

### Experimental design

We applied the nutrient treatments five weeks after transplantation by injecting 9 aliquots of 2 mL solution inside the buried ring using a template placed on the soil surface to assure an even distribution of nutrients and minimal loss of the solution. We added 6.5 μg and 65 μg ^15^NH_4_^+^-N (99 atom percent) g^-1 ^dry soil in the ring for the low and high NH_4_^+ ^treatments, respectively (1.35 mg ^15^NH_4_^+^-N per ring and 13.5 mg ^15^NH_4_^+^-N per ring, respectively). Water was used as a control. Each treatment consisted of three biological replicates, and plants were destructively harvested at 5, 29, 53, and 96 hrs after ^15^NH_4_^+^-N addition, for a total number of 36 plants in an unreplicated block design. The transcriptome analyses were performed on roots harvested at 53 hrs post-treatment.

### Harvest and sample analysis

At harvest, shoots were severed at the soil surface, dried at 60°C, weighed, and ground to a fine powder for isotope analyses. Immediately following the harvest of the aboveground biomass, the root in-growth rings were carefully exposed, and the roots growing into and out of the ring were severed. Patch roots for transcriptome analysis were rinsed and immediately frozen in liquid nitrogen. A homogenous sub-sample of the patch soil and the surrounding pot soil was immediately removed for gravimetric water content and soil inorganic N concentrations. Soil NH_4_^+ ^and NO_3_^- ^were analyzed after KCl extractions and colorimetric determination using modifications of Miranda *et al.*, [61] and Foster *et al.*, [62] respectively. A small subsample of ring roots was scored for arbuscular mycorrhizal fungi at 200× [63]. The remaining patch roots were washed by wet sieving, dried at 60°C, weighed, and ground to a fine powder for isotope analyses. All dried plant material was analyzed for δ^15^N on a PDZ Europa ANCA-GSL elemental analyzer and a PDZ Europa 20-20 isotope ratio mass spectrometer (Sercon Ltd., Cheshire, UK) at the UC Davis Stable Isotope Facility, USA. Background ^15^N was calculated as the average atom percent ^15^N in the water samples (mean atom percent ^15^N ± SD in shoots: 0.369% ± 0.0003). Leaf total P was analyzed by microwave digestion with nitric acid/hydrogen peroxide [64] followed by atomic absorption spectrometry and inductively coupled plasma atomic emission spectrometry at the UC Davis Division of Agriculture and Natural Resource Laboratory.

### Soil analysis

Soil nutrient and plant isotope data were analyzed with a two-way analysis of variance (ANOVA) with harvest time, nutrient addition, and block as fixed main effects. All two-way interactions were tested. The three way interaction was not tested because of insufficient degrees of freedom. Data was checked to assure that the ANOVA assumptions were met and was transformed as necessary. Tukey-Kramer Honestly Significant Difference test was used to determine differences between means at P < 0.05. All data were analyzed using R (R Core Development Team 2007).

### RNA isolation

Root RNA samples were extracted using the RNeasy Plant Mini Kit (Qiagen Sciences, Germantown, MD, USA) following the manufactures guidelines plus a third wash step before elution. RNA concentrations and quality were assessed using the Agilent Nanodrop and the RNA 6000 Nano Assay (Bioanalyzer 2100, Agilent, Santa Clara CA). RNA samples had RNA integrity numbers (RIN) of at least 7.0. DNase digestion was performed on 20 ug total RNA using RQ1 RNase-free DNase (Promega, Madison WI). These RNA were used for both microarray analysis and cDNA synthesis for qRT-PCR analysis.

### Microarray analysis

Transcriptome profiling of each 53h-post injection RNA sample was performed using the Tomato Genome Array Chip (Affymetrix, Santa Clara, CA, USA). RNA samples were prepared for microarray analysis using the MessageAmp Premier RNA Amplification Kit (Ambion, Foster City CA) with 200 ng total RNA as input. Fragmented cRNA samples were then sent to the University of Missouri's DNA Core Facility for Array hybridization and scanning. Each array's CEL file was summarized in Affymetrix Expression Console software using the MAS5 algorithm. The signal intensities were log transformed, and quality control analysis performed. This array data has been made available on the Gene Expression Omnibus (GEO; http://www.ncbi.nlm.nih.gov/projects/geo/) accession #GSE21020. The data were filtered to remove probesets whose log signal intensity was below 4.605 in all 9 arrays. For each probeset, which represents the combined expression data from all relevant probe pairs on the chip, the generalized linear model Y_ij _= μ + T_i _+ ε_ij _was fit. In each ANOVA, Y_ij _is the log normalized transcript level for the i^th ^treatment and the j^th ^replicate, μ is the overall mean expression for the probeset and Ti represents the i^th ^treatment (water, low nutrient, and high nutrient). The null hypothesis t_1 _= t_0 _(i.e., mean expression not different between a pair of treatments) was tested using an F-test. We examined the model for conformation to the assumption of normality of the residuals testing the null hypothesis that the residuals for each gene were normally distributed using the Shapiro-Wilk Test. All analyses were performed in JMP Genomics 3.0 (SAS Institute, Cary NC). An FDR level of 10% was used for declaring findings significant, and a stringent rate of 5% was also examined [65,66]. ANOVA analysis of all differentially expressed genes can be found in Additional file 1. The list of statistically significant transcripts was initially annotated using the Affymetrix NetAffyx annotation file to match a representative Genbank public ID and Unigene to each probeset. The functional annotations of the Unigenes were grouped into functional categories as described in Bevan *et al. *[67]. In cases where the tomato gene did not have a matching Unigene or had not been functionally annotated, the tomato sequence was used to identify Arabidopsis orthologues by WU-BLAST searches at TAIR [68]. To test for enrichment of specific functional categories between the high vs water and low vs. water pairwise comparisons, Fisher's exact test was performed using GraphPad Prism 5.0, San Diego CA). Within specific categories, binomial distribution probability tests were performed to test for enrichment in the up/down regulation patterns of functionally related genes (expectation = 0.5).

### Quantitative real-time RT-PCR

cDNA was synthesized from 1.5 ug DNase-treated total RNA using the Superscript III kit (Invitrogen Carlesbad CA). Gene-specific primer sets were designed using IDT's primerquest software program (Additional file 3), and their sequence uniqueness confirmed with a nucleotide BLAST search against the tomato genome database. Primer pairs were tested for specificity and efficiency with serial dilution reactions and dissociation curve analysis post-amplification. Real-time PCR reactions were run on the Stratagene MX3000 PCR machine using Sybr Green chemistry (Invitrogen Platinum Sybr Green II master mix, 400 nM primer concentration, ROX reference dye, and 1:150 diluted cDNA). Multiple reference control genes were tested against all samples to identify control genes whose expression was not affected by the NH_4_^+ ^treatments. *LeACT *and *LeUBI *were similarly expressed across the samples while *LeTubulin *was differentially expressed. *LeACT *was subsequently used as the reference control gene, and the relative expression of the various target genes was analyzed according to the ΔΔCT method [69]. Standard error was computed from the average of the ΔCT values for each biological sample [70].

## Authors' contributions

DRR carried out the microarray and molecular genetic studies, participated in the statistical analyses, and drafted the manuscript. FB-M and NTH participated in the greenhouse experiments and nutrient analyses, and helped write the manuscript. LEJ and DPS designed and coordinated the study, participated in the greenhouse experiments and data analyses, and helped write the manuscript. All authors read and approved the final manuscript.

## Supplementary Material

Additional file 1 Microarray analysis of differentially expressed genes across all treatments including the Affymetrix probeset ID, Genbank public ID, linear fold changes, FDR P-values, gene annotation information, and probeset redundancy information. Click here for file

Additional file 2 Genes similarly regulated by high NH_4_^+ ^and low NH_4_^+ ^treatments compared to the water control. Click here for file

Additional file 3 Primer sequences for genes assayed with qRT-PCR. Click here for file
